# High background rates of positive tuberculosis-specific interferon-γ release assays in a low prevalence region of UK: a surveillance study

**DOI:** 10.1186/1471-2334-12-339

**Published:** 2012-12-06

**Authors:** Timothy SC Hinks, Nimu Varsani, David T Godsiff, Thomas C Bull, Katherine L Nash, Lisa McLuckie, Catherine Maule, Tessa Flower, Anthony Warley

**Affiliations:** 1 Academic Unit of Clinical and Experimental Sciences, Mailpoint 0810, University of Southampton Faculty of Medicine, Southampton General Hospital, Southampton, SO16 6YD, UK; 2 Salisbury NHS Foundation Trust, Salisbury District Hospital, Salisbury, Wiltshire, SP2 8BJ, UK; 3 St George’s, University of London, Cranmer Terrace, London, SW17 0RE, UK

**Keywords:** Tuberculosis, Diagnosis, Mycobacterium, T-Spot.*TB*, Enzyme-linked immunospot

## Abstract

**Background:**

Background rates of latent tuberculosis infection in low prevalence regions of Britain are unknown. These would be valuable data for interpreting positive IGRA results, and guiding cost-benefit analyses. The management of a large outbreak of tuberculosis occurring in a rural district hospital provided an opportunity to determine the background rates and epidemiology of IGRA-positivity amongst unselected hospital patients in a low-prevalence region of U.K.

**Methods:**

As part of a public health surveillance project we identified 445 individuals exposed to the index cases for clinical assessment and testing by a TB-specific interferon-γ release assay (IGRA): T-Spot.*TB*. Uniquely, an additional comparator group of 191 age-matched individuals without specific recent exposure, but with a similar age distribution and demographic, were recruited from the same wards where exposure had previously occurred, to undergo assessment by questionnaire and IGRA*.*

**Results:**

Rates of IGRA positivity were 8.7% (95%CI, 4.2-13, *n*=149) amongst unexposed patients, 9.5%(3.0-22, *n*=21) amongst unexposed staff, 22%(14–29, *n*=130) amongst exposed patients, 11%(6.1-16, *n*=142) amongst exposed staff. Amongst the individuals without history of recent exposure to the outbreak, IGRA-positivity was associated with prior TB treatment (OR11, P.04) and corticosteroid use (OR5.9, P.02). Background age-specific prevalences of IGRA-positivity amongst unexposed individuals were: age <40 0%(N/A), age 40–59 15%(12–29), age 60–79 7.0%(1.1-13), age≥80 10%(5.9-19).

**Conclusions:**

Background rates of IGRA-positivity remain high amongst unselected white-Caucasian hospital inpatients in U.K. These data will aid interpretation of future outbreak studies. As rates peak in the 5^th^ and 6^th^ decade, given an ageing population and increasing iatrogenic immunosuppression, reactivation of LTBI may be a persistent hazard in this population for several decades to come.

## Background

Background rates of latent tuberculosis infection (LTBI) in low prevalence regions of Britain are unknown. Current estimates depend on extrapolations from the incidence of active cases of TB [1], or on tuberculin skin testing [2], which is confounded by vaccination. TB-specific interferon-γ release assays (IGRAs) are sensitive and specific methods for detecting immune sensitisation by TB [3], and have accurately characterised the epidemiology of LTBI amongst high risk populations such as recent immigrants or TB contacts. The few studies which have reported on IGRA-positivity in healthcare workers place rates in the range of 6.7-9.9% in low prevalence countries [4]. However there is a paucity of data on the background rates of IGRA-positivity in low risk populations, such as hospital in patients without recent TB exposure [1]. Such data would be valuable for interpreting the likely clinical significance of a positive IGRA result in low risk populations, and further informing cost-benefit analyses of new diagnostics [1].

We undertook to manage an outbreak in a low prevalence region by contact tracing with standard IGRA testing according to the “stone in the pond” principle [1], and also to determine the background rates and epidemiology of IGRA-positivity amongst unselected, unexposed hospital patients in a low-prevalence region of U.K.

## Methods

**Figure 1:**
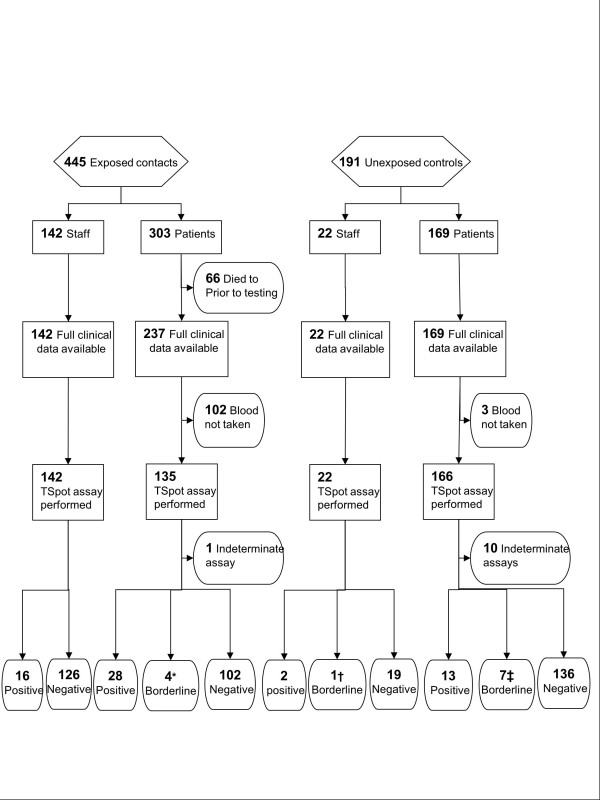
**Study flow chart showing all participant cohorts.** * comprises 3 borderline positive, 1 borderline negative; † comprises 1 borderline negative; ‡ comprises 5 borderline positive, 2 borderline negative.

In June 2009 a patient was diagnosed with smear-positive pulmonary tuberculosis over three weeks after admission to Salisbury District Hospital, a rural district general hospital in South West England. Over the subsequent 15 months a further 8 secondary cases of active TB were diagnosed amongst staff and patients. 6/6 culture-confirmed cases had identical VNTR (variable number tandem repeat) profile, so were directly attributable to the index case, given the very low incidence of active TB locally at 6.1/100,000/year [5]. Contact tracing identified 445 potential contacts of the index case, comprising 142 staff and 303 patients. These two groups of “exposed staff” and “exposed patients” were defined by having received treatment or worked regularly on the same wards at the same time that the index case was present, and were therefore considered to have had known, recent exposure to MTB. “Exposed staff” had a median age of 34 (range 18 to 69) and 84% were BCG vaccinated. “Exposed patients” were older with a median age of 73 (range 27 to 95) and only 41% were known to be BCG vaccinated. See Table 1 and Figure 1.

**Table 1:** Demographics and T-Spot results of all participants

	**Exposed**						**Unexposed**					
	**Staff**			**Patients**			**Staff**			**Patients**		
	**n=142**			**n=134**			**n=22**			**n=156**		
	**n**	**%**	**(95% CI)**	**n**	**%**	**(95% CI)**	**n**	**%**	**(95% CI)**	**n**	**%**	**(95% CI)**
Age (years), median (range)	34	(18 to 69)		73	(27 to 95)		44	(23 to 60)		71	(25 to 93)	
Male, n (%)†	25	(17)		60	(45)		7	(32)		77	(49)	
Ethnicity, n (%)												
White Caucasian	-	-		133	(99)		20	(91)		156	(100)	
Hispanic	-	-		1	(0.7)		0	(0)		0	(0)	
Asian‡	-	-		0	(0)		2	(9)		0	(0)	
BCG (history or scar), n (%)												
Yes	119	(84)		55	(41)		18	(82)		75	(48)	
No	8	(5.6)		57	(43)		3	(14)		50	(32)	
Unknown	1	(0.7)		22	(16)		1	(5)		30	(19)	
TSpot result including initial borderline results, n, (%) (95%CI)												
Positive	14	(9.9)	(5.0 to 15)	28	(21)	(14 to 27)	2	(9.1)	(0 to 21)	13	(8.3)	(4.0 to 13)
Borderline positive	2§	(1.4)	(0 to 3.3)	3	(2.2)	(0 to 4.7)	0	(0)	(N/A)	5	(3.2)	(0.44 to 6.0)
Borderline negative	6§	(4.2)	(0.92 to 7.5)	1	(0.7)	(0 to 2.2)	1	(4.5)	(0 to 13)	2	(1.3)	(0 to 3.0)
Negative	120	(85)	(79 to 90)	102	(76)	(68 to 82)	19	(86)	(72 to 100)	136	(87)	(82 to 92)
TSpot result excluding those borderline results which were not retested, n, (%) (95%CI)												
Positive	16	(11)	(6.1 to 16)	28	(22)	(14 to 29)	2	(9.5)	(0 to 22)	13	(8.7)	(4.2 to 13)
Negative	126	(89)	(84 to 94)	102	(78)	(71 to 85)	19	(90)	(78 to 100)	136	(91)	(87 to 96)

† Percentages are those of those with valid data (e.g. occupation).

‡ India (1), Philippines (1).

§ Borderline results were retested and definitive results reached in every case. 2 (of which one was borderline negative) became positive (conversions), 6 (of which one was borderline negative) became negative (reversions).

Uniquely, to inform interpretation of positive results obtained amongst the exposed contacts, two additional comparator groups of “unexposed” individuals were recruited. These comprised a further 191 individuals with a similar age distribution recruited from staff (“unexposed staff”, n=22) and adult patients (“unexposed patients”, n=169), see Figure 1. These individuals were recruited from the same respiratory and general medical wards where exposure had previously occurred, but who had not been exposed to any of the 9 cases at the time of their admissions. Subjects with any suspected TB exposure within the last 2 years were excluded. Demographics of the unexposed individuals are shown in Table 2. “Unexposed staff” had a median age of 44 (range 23 to 95) and 82% were BCG vaccinated. “Unexposed patients” were older with a median age of 71 (range 25 to 93) and only 48% were known to be BCG vaccinated. This sample size was predicted to give a confidence interval width for overall LTBI prevalence of <4.5%.

**Table 2:** Demographics of the unexposed individuals

		**Unexposed, age-matched individuals**					
		**TSpot positive n=13**		**TSpot negative n=136**		**Odds ratio**	**Univariate P**
		**n**	**%**	**n**	**%**		
Age (years), median (range)		64	(45-87)	70	(25-93)		0.81
Male, n (%)		5	(38)	70	(52)		0.36
Ethnicity, n (%)							
White Caucasian		13	(100)	136	(100)		
Country of birth							
UK born		9	(69)	126	(93)	**0.18**	**0.006**
Other		4	(31)†	10	(7.4)		
Years since immigration, median (range)		43	(21-62)	52	(8-61)		
Ever visited a high prevalence country, n (%)		4	(31)	47	(35)		0.84
Years since last visit, median (range)		8.5	(0-40)	5.5	(0-60)		
Ever resident in a high prevalence area >6/52, n (%)		2	(15)	27	(20)		0.64
Occupation: healthcare, prison, lab n (%)‡		4	(31)	18	(13)		0.09
BCG (history or scar), n (%)							
Yes		7	(54)	66	(49)		0.73
No		4	(31)	44	(33)		
Unknown		2	(15)	26	(19)		
Medical history, n (%)							
Comorbidity known to be associated with TB§		0	(0)	21	(16)		0.22
Immunosuppresive medications∥		4	(31)	12	(9.2)	**4.6**	**0.015**
Ever known to be exposed to TB		4	(31)	26	(19)		0.32
Ever had treatment for tuberculosis		2	(15)	2	(1.5)	**12**	**0.003**

† Germany (2), South Africa (1), Hong Kong (1).

‡ Percentages are those of those with valid data.

§ Diabetes mellitus (17), chronic renal failure (3), haematological malignancy (2), gastric surgery (1).

∥ Systemic steroids (12), methotrexate (4), hydroxychloroquine (2), anti-TNF alpha (2), sulfasalzine (1).

Participants were invited for screening by history and IGRA. Samples were couriered to Oxford Immunotec for testing by T-Spot.®*TB*. (Oxford Immunotec, Oxford, U.K.) A positive result comprised ≥8 spot-forming colonies (SFC) more than the control well. A result of 5, 6 or 7 SFC was considered borderline and the subject retested where possible, or removed from subsequent analysis. 95% confidence intervals and multivariate binary logistic regression (at *P*<0.05), were calculated using SPSS version 19.0 (IBM Corp., Armonk, NY).

Subjects provided written consent. As a primarily public health surveillance exercise the National Research Ethics Service advised this study did not require review by a NHS Research Ethics Committee.

## Results

Full clinical data were available and IGRA performed on 465 staff and patients. Study design and assay results are shown Figure 1. Demographic data for the complete investigation are presented in Table 1. All 142 exposed staff completed screening. Amongst the group of exposed patients, testing was not performed in 168 exposed patients because they had died (n=66), or (n=102) testing was declined due to patient preference or considered inappropriate, for example due to instigation of end of life care. Three unexposed patients withdrew consent. Overall 11 assays were indeterminate because of insufficient cells (n=9) – predominantly due to under-filled tubes – or high background (n=2).

The rates of IGRA positivity were: unexposed patients 8.7%(95%CI, 4.2-13, n=13/149), unexposed staff 9.5%(95%CI, 3.0-22, 2/21), exposed patients 22%(95%CI, 14–29, 28/130), exposed staff 11%(95%CI, 6.1-16, 16/142).

From these data we estimate an additional 35/379 living staff (n=142) and patients (n=237) known to be exposed during this outbreak, would test positive by IGRA, if full data were available. These excess positive IGRAs probably reflect recently-acquired LTBI.

**Figure 2:**
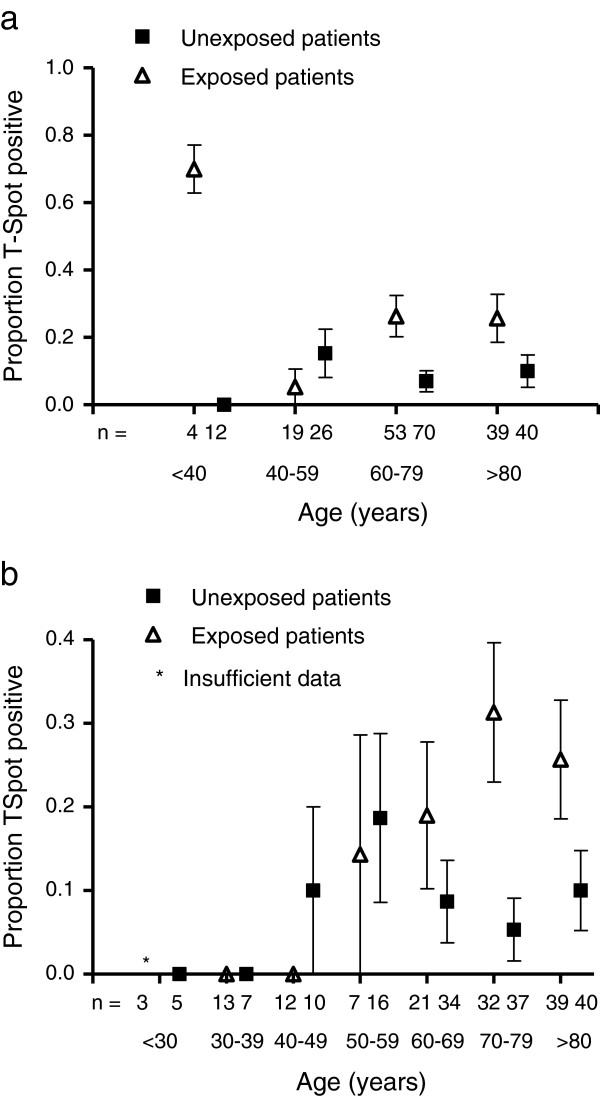
**Age specific prevalences of IGRA-positivity amongst exposed patients and amongst unexposed patients.** Error bars show standard errors of the mean. **a**) data stratified according to four age-groups. **b**) data stratified by seven age groups, as per Syed *et al.*[2].

Amongst unexposed patients positive IGRA results were positively associated in multivariate analyses with history of previous TB treatment (OR 11, P=.04) and with use of corticosteroids (OR 5.9, P=.02). 6/13 IGRA-positive unexposed patients had a history of TB treatment (n=2), definite (n=2) or probable (n=2) TB exposure. Age-specific prevalences of IGRA-positivity amongst all unexposed patients were: age <40 0%(95%CI N/A), age 40–59 15%(95%CI 12–29), age 60–79 7.0%(95%CI 1.1-13), age≥80 10%(95%CI 5.9-19) (Figure 2a,b).

## Discussion

We observed a background rate (8.7%) of IGRA-positivity amongst an unselected population typical of respiratory and general medical inpatients in a rural district hospital in U.K. All these “unexposed” patients, without known recent TB exposure, were white-Caucasians, a population in whom data on LTBI prevalence are lacking [2], but who comprise 92% of the U.K. population [6]. As the South West region has the lowest incidence of active tuberculosis in mainland Britain [5], and as our findings accord with a 9.0% rate in a similar study using QuantiFERON®-TB in Northern Ireland [7], this may represent a current minimum U.K. background rate of IGRA-positivity.

Rates of IGRA-positivity amongst unexposed control patients were higher in subjects receiving long term iatrogenic immunosuppression (OR 5.9). This would be consistent with the known increased risk of acquiring MTB-complex infection due to impairment of cell-mediated immunity induced by therapy with corticosteroids or with anti-TNFα therapy [8].

The 9.5% rate of IGRA-positivity amongst the unexposed staff is consistent with a rate of 9.9% observed by Schablon *et al.* in their large study of healthcare workers in Germany [4]*.* These authors observed associations between IGRA positivity and older age, foreign birth and prior personal history of TB. In our study the IGRA positive, unexposed staff were neither foreign born nor had a personal history of TB, but were both in their 6^th^ decade. It seems likely that relatively high rates of IGRA-positivity in healthcare workers may be due to unrecognised occupational exposure to MTB, particularly in older workers whose exposure may predate current infection control procedures.

Interestingly the age-specific prevalence amongst unexposed controls shows a similar bimodal distribution to that observed in the early 1990s in Liverpool [2] using the Heaf method. The lower absolute values reflect the greater specificity of T-Spot.*TB* and the declining rates of TB amongst U.K. born individuals [9-11]. It is estimated that the annual risk of infection in U.K. declined from 12% in 1901 to 1.9% in 1949 [10] and has since fallen further [11]*.* Nonetheless our data suggest there is still a peak in the 5^th^ and 6^th^ decades, as observed two decades ago [2].

Do these positive IGRAs represent true LTBI? A positive T-Spot.*TB* assay is evidence of previous immune sensitisation by specific antigens from *M.tb* complex [3,12]. 6/13 IGRA-positive individuals without recent exposure had a known history of prior TB infection or exposure. That T-Spot responses wane over time [13], or may be transiently positive [3], suggests some individuals might spontaneously clear *M*.*tb* infection. Alternatively, as early secretory antigenic target 6 and culture filtrate protein 10 are expressed by other opportunistic mycobacteria [14], and indeed QuantiFERON responses have been observed with *M.marinum, M.kansasii,* and *M.szulgai*[12] some positive IGRA results might be attributable to other opportunistic mycobacteria, or even to unpasteurised dairy products [7], or the *M.bovis* epidemic affecting more than 5% of cattle herds locally [15], although we found no association with farming occupation (Data not shown).

This study has several potential limitations: sample sizes are relatively small, particularly amongst younger age-groups; data may not be directly generalizable to other regions or non-hospital communities; due to the time-course of the outbreak, sample sizes and use of chemoprophylaxis, the study was not designed to provide data on rates of progression amongst IGRA positive individuals.

## Conclusions

8.7% of unselected white-Caucasian medical inpatients in a low-prevalence region test positive by T-Spot. As this region has the lowest UK incidence of active TB, this figure may represent a current minimum UK background prevalence of LTBI. These data will aid interpretation of future outbreak studies. They will also inform cost-benefit analyses which may be sensitive to assumed background rates of LTBI. As rates peak in the 5^th^ and 6^th^ decade, given an ageing population and increasing iatrogenic immunosuppression, reactivation of LTBI may be a persistent hazard in this population for several decades to come.

## Abbreviations

TB: Tuberculosis; LTBI: Latent tuberculosis infection; IGRA: Interferon-γ release assay; VNTR: Variable number tandem repeat; SFC: Spot-forming colonies.

## Competing interests

The authors declare they have no competing interests.

## Authors’ contributions

TH helped design the study, analysed the data and drafted the manuscript. NV DG TB KN LM CM TF collected data. AW conceived the study and participated in its design and coordination. All authors read and approved the final manuscript.

## Pre-publication history

The pre-publication history for this paper can be accessed here:

http://www.biomedcentral.com/1471-2334/12/339/prepub
